# Neural-Discursive Dynamics in Collaborative Spatial Problem Solving: Evidence from fNIRS Hyperscanning and Accountable Talk

**DOI:** 10.3390/bs16101787

**Published:** 2026-10-01

**Authors:** Qi Zeng, Yi Liu, Ziyu Liu, Xingguang Guo

**Affiliations:** Faculty of Education, Beijing Normal University, Beijing 100875, China

**Keywords:** collaborative problem solving, accountable talk, inter-brain synchrony, fNIRS hyperscanning, multimodal learning analytics, task performance

## Abstract

High-quality collaboration cannot be inferred simply from how much students talk or how strongly their brain activity is synchronized during joint work. This study examined relations among accountable talk, inter-brain synchrony (IBS), and group task performance in fifth-grade students’ collaborative mathematical problem solving. Forty-nine students worked in 15 groups and completed two collaborative spatial design tasks, yielding 30 group × task records. Functional near-infrared spectroscopy (fNIRS) data and video recordings were collected simultaneously; discourse was coded using accountable talk indicators, and group design products were scored as task performance. Mixed-effects models showed that higher task performance was associated with more accountable talk turns, shorter mean turn duration, and higher accountable talk density, but not with longer total accountable talk duration. IBS showed channel-group-specific associations with task performance, suggesting that overall synchrony alone did not fully characterize collaborative activity. Temporal-window analyses showed that the within-window association between accountable talk density and IBS differed according to task performance level. These findings suggest that, within the present collaborative tasks, productive collaboration was associated with dense task-relevant accountable discourse and performance-dependent behavioral–neural associations rather than talk quantity or overall neural synchrony alone.

## 1. Introduction

Collaborative learning is widely used to support students’ engagement with complex and open-ended problems. However, the quality of collaboration cannot be inferred simply from how much students talk or how active their interaction appears to be. In small-group problem solving, students need to transform individual ideas into shared, examinable, and revisable resources through dialogue, while also coordinating attention, reasoning, and action during the task. Research in social neuroscience and multimodal learning analytics suggests that such coordination can be examined through observable interactional processes as well as through inter-brain synchrony during joint activity. Yet less is known about how task-relevant accountable discourse and inter-brain synchrony are temporally associated in classroom-like collaborative problem solving, or whether this association differs across groups with different levels of task performance. The present study addressed this issue in the context of elementary students’ collaborative spatial design tasks by examining accountable talk indicators, channel-group-specific inter-brain synchrony, and their within-window association at the temporal-window level.

### 1.1. Collaborative Learning as Coordinated Problem Solving

Complex problem solving often requires students to organize individual ideas into a shared plan that can be developed, tested, and revised. The mere presence of a group does not indicate that collaborative learning has occurred. Rather, collaborative learning is better understood as a process in which learners engage with a joint task, respond to one another, and construct meaning through interaction (Dillenbourg, 1999). In the collaborative spatial design tasks examined in this study, students had to coordinate multiple constraints, including area, location, function, pathways, and spatial relations. Such open-ended mathematical problem solving requires students not only to work on a complex task but also to sustain a collaborative process through which their solution can be progressively advanced (Ceballos et al., 2026).

A central issue in this process is how students treat one another’s ideas. Barron (2003) showed that potentially useful ideas do not automatically become resources for group problem solving; they become consequential only when peers acknowledge, respond to, evaluate, and develop them. Recent work on collaborative dialogue and dialogue productivity similarly emphasizes that productive collaboration depends on students’ ability to share ideas, listen and respond to others, engage with peers’ reasoning, and regulate the joint task through negotiation (de Araujo et al., 2025; Kim et al., 2026). Thus, the key question is not whether students speak frequently, but whether their interaction enables individual ideas to become shared resources for problem solving.

### 1.2. Accountable Talk as a Behavioral Indicator of Productive Collaboration

Accountable talk refers to task-relevant discourse through which students make their contributions accountable to the learning community, to knowledge and evidence, and to standards of reasoning. It emphasizes that students should not only express opinions but also respond to others, provide evidence, explain reasons, and evaluate ideas during collaborative activity (Michaels et al., 2008). This orientation is consistent with research on productive interaction in collaborative problem solving, where effective dialogue depends on students’ ability to explain ideas, take up peers’ reasoning, and move emerging ideas toward shared evaluation and revision (Webb et al., 2021).

In the present study, accountable talk was operationalized as coded conversational turns that performed task-relevant accountable discourse functions during collaborative spatial design. These accountable talk turns included proposing design ideas, adding task-relevant information, responding to peers, evaluating the reasonableness of solutions, explaining spatial relations, and revising the design. This operationalization treats accountable talk as a set of discourse indicators rather than as a general label for all classroom talk.

However, the role of accountable talk cannot be evaluated only by whether it occurs or by how often it occurs. Productive dialogue is sustained across sequences of interaction rather than appearing only as isolated turns (de Araujo et al., 2025; Hu, 2021). In collaborative spatial design, a single long utterance may not necessarily advance the group solution more effectively than several short, timely proposals, responses, evaluations, and revisions. For this reason, the present study examined not only the number of accountable talk turns but also the total duration of accountable talk, the average duration of accountable talk turns, accountable talk density per unit of speaking time, and the temporal distribution of accountable talk density across the task. This allowed us to examine whether higher-performing groups were characterized by more or longer accountable talk, or instead by a denser, more compact, and more timely organization of task-relevant accountable discourse.

### 1.3. Inter-Brain Synchrony in Collaborative Learning and Teamwork

In addition to observable discourse, collaboration can also be examined through coordinated changes in group members’ neural activity. Functional near-infrared spectroscopy (fNIRS) hyperscanning enables researchers to record brain activity from multiple interacting participants simultaneously and to examine inter-brain synchrony (IBS) during joint tasks. IBS should not be interpreted as a direct measure of learning, understanding, or collaborative activity. Rather, IBS provides a complementary index of cross-participant covariation in neural signals during shared activity and should be interpreted alongside task and behavioral evidence.

Previous studies suggest that IBS can be associated with shared understanding, active discussion, and task performance. A systematic review and meta-analysis of fNIRS hyperscanning studies found that cooperative behavior was consistently associated with inter-brain synchrony in frontal and temporoparietal regions, particularly the prefrontal cortex (Czeszumski et al., 2022). For example, Pan et al. (2023) found that triadic collaborative learning showed higher within-group neural synchrony than individual learning, and that this synchrony was stronger when groups reached consensus and was associated with learning outcomes. Azhari et al. (2025) similarly found that IBS emerged mainly during active discussion in naturalistic online seminars and was related to task performance. These findings indicate that IBS may provide a complementary source of evidence for understanding collaborative processes in learning tasks.

At the same time, higher IBS should not be assumed to indicate better collaboration. Zhang et al. (2024), in a collaborative tangram problem-solving study, found that division-of-labor collaboration led to better behavioral performance, whereas more collaboration increased IBS. This suggests that stronger synchrony does not necessarily correspond to better task outcomes. Froese et al. (2024) also argued that both synchrony and desynchrony may have functional significance, depending on the task, interactional process, and neural system involved. Therefore, the present study did not assume that higher overall IBS necessarily reflects higher-quality collaboration. Instead, it distinguished IBS across functional fNIRS channel groups and examined whether channel-group-specific IBS was associated with group task performance.

### 1.4. From Parallel Indicators to Temporal Behavioral–Neural Associations

Analyzing accountable talk alone can show how students advance a shared solution through proposals, responses, explanations, evaluations, and revisions. However, as an observable discourse indicator, accountable talk by itself cannot fully characterize the neural activity observed during these task-relevant contributions (Cukurova et al., 2020; Michaels et al., 2008). Conversely, analyzing IBS alone can identify patterns of inter-brain synchrony during a joint task, but IBS is not self-explanatory. Its meaning needs to be interpreted in relation to the task context and behavioral evidence (De Felice et al., 2025; Hakim et al., 2023). Combining accountable talk density with IBS therefore makes it possible to move beyond examining task-relevant accountable discourse and inter-brain synchrony as separate indicators. Instead, it allows us to investigate temporal associations between observable task-relevant accountable discourse and inter-brain synchrony during collaborative activity.

This behavioral–neural association also needs to be examined at the temporal-window level. Collaborative spatial design is not a homogeneous process. It involves multiple phases, such as understanding the task, coordinating constraints, generating a solution, drawing the design, and organizing the final response. Analyses based only on whole-task frequencies or averages may therefore obscure temporal variation in discourse and neural synchrony across different task segments. Multimodal research on collaborative learning has shown that temporal features can provide information beyond aggregate counts or averages when interpreting collaborative processes and learning outcomes (Olsen et al., 2020). Recent studies have also emphasized that language, gesture, gaze, and bodily action vary over time and are interrelated in collaborative learning, making static summary measures insufficient for characterizing process-level variation (Yan et al., 2025). This temporal perspective is further supported by educational neuroscience evidence suggesting that the learning relevance of brain-to-brain synchrony may also be temporally localized, with higher synchrony observed during instructional segments linked to questions that students subsequently answered correctly (Davidesco et al., 2023).

Existing studies have begun to address different components of this temporal behavioral–neural relationship. Dynamic IBS analyses have shown that inter-brain synchrony varies over the course of real collaboration (Li et al., 2021), while recent work has begun to examine associations between dialogue content and interpersonal neural synchrony in the prefrontal cortex (Carollo et al., 2025). However, relatively little is known about whether temporal variation in accountable talk density is associated with channel-group-specific IBS within the same windows in classroom-like collaborative problem solving. Even less is known about whether the strength of this behavioral–neural association varies according to group task performance. The core gap addressed in this study is therefore whether accountable talk density and channel-group-specific IBS show within-window associations, and whether the strength of these associations differs according to group task performance.

### 1.5. The Present Study

The present study examined the relations among accountable talk, inter-brain synchrony, and group task performance in elementary students’ open-ended collaborative spatial design tasks. Students worked in groups of three or four to complete a spatial design problem. We synchronously collected fNIRS data and video recordings, coded students’ dialogue using accountable talk indicators, and used scores on the group spatial design products as the measure of task performance.

Building on the gaps identified above, the study integrated accountable talk, channel-group-specific IBS, and task performance within a process-oriented analytic framework. Specifically, it examined whether higher-performing groups showed denser task-relevant accountable discourse, whether IBS in different functional fNIRS channel groups was associated with task performance, and whether the within-window association between accountable talk density and IBS varied according to group task performance.

The following research questions guided the study:

RQ1. Do groups with higher task performance show different accountable talk characteristics?

RQ2. Is inter-brain synchrony functionally channel-group-specific, and how is channel-group-specific synchrony associated with task performance?

RQ3. Is accountable talk density associated with inter-brain synchrony at the temporal-window level, and does this association vary according to task performance?

Together, these questions examine whether differences in group task performance are associated not simply with students talking more, speaking for longer, or showing higher overall neural synchrony, but with a temporally organized collaborative process in which dense task-relevant accountable discourse is associated with channel-group-specific inter-brain synchrony patterns during collaborative problem solving.

## 2. Materials and Methods

### 2.1. Participants and Study Design

This study used a group-level multimodal process analysis design. The participants were 49 fifth-grade students with a mean age of 10.5 years (*SD* = 0.69), including 24 girls. The students were recruited from multiple schools, and students in the same testing session were not previously acquainted with one another. All participants were right-handed, had no reported neurological disorders, and had acquired the basic mathematical knowledge needed to complete the tasks, including rectangular area and decimal operations.

Fifth grade was selected as an appropriate developmental and curricular stage for this study. Students at this stage have accumulated experience with small-group collaboration and are generally capable of engaging in sustained discussion, perspective coordination, and joint reasoning. Moreover, the spatial design tasks required mathematical knowledge including area, quantity relations, and decimal operations, which aligns with the curriculum expectations for upper elementary students.

The final sample included 15 groups, comprising eleven three-student groups and four four-student groups. Groups were formed by balancing Raven’s reasoning test scores, Big Five personality scores, and gender composition to make the groups broadly comparable at baseline. Each four-student group included two boys and two girls. Each three-student group had either a 1:2 or 2:1 gender ratio.

Each group completed two parallel open-ended collaborative spatial design tasks with comparable structural demands in sequence. The apartment spatial design task was administered first, followed by the campus sports event task. The fixed sequence was adopted to maintain a consistent experimental procedure across groups and standardized data collection conditions. The final analytic dataset therefore contained 30 group × task records. The study was approved by the Research Ethics Review Committee of the Faculty of Education, Beijing Normal University (Approval No. BNU202210100038; 10 October 2022). Written informed consent was obtained from parents or legal guardians, and written assent was obtained from all participating students before data collection.

### 2.2. Collaborative Spatial Design Tasks and Multimodal Data Collection

Both tasks were open-ended collaborative spatial design tasks. Students were asked to develop a reasonable spatial layout plan based on given spatial conditions and functional requirements; a representative task description and the original spatial boundary diagram for the apartment spatial design task are provided in Supplementary Text S1 and Supplementary Figure S1, respectively. The two task versions shared comparable structural demands, including the interpretation of spatial constraints, coordination of multiple design requirements, and generation of a collaborative design solution (Supplementary Table S1). The tasks involved area, location, functional zoning, circulation, and coordination of multiple constraints. Students therefore needed to generate, evaluate, and revise their design solutions through group discussion after individual thinking.

**Figure 1:**
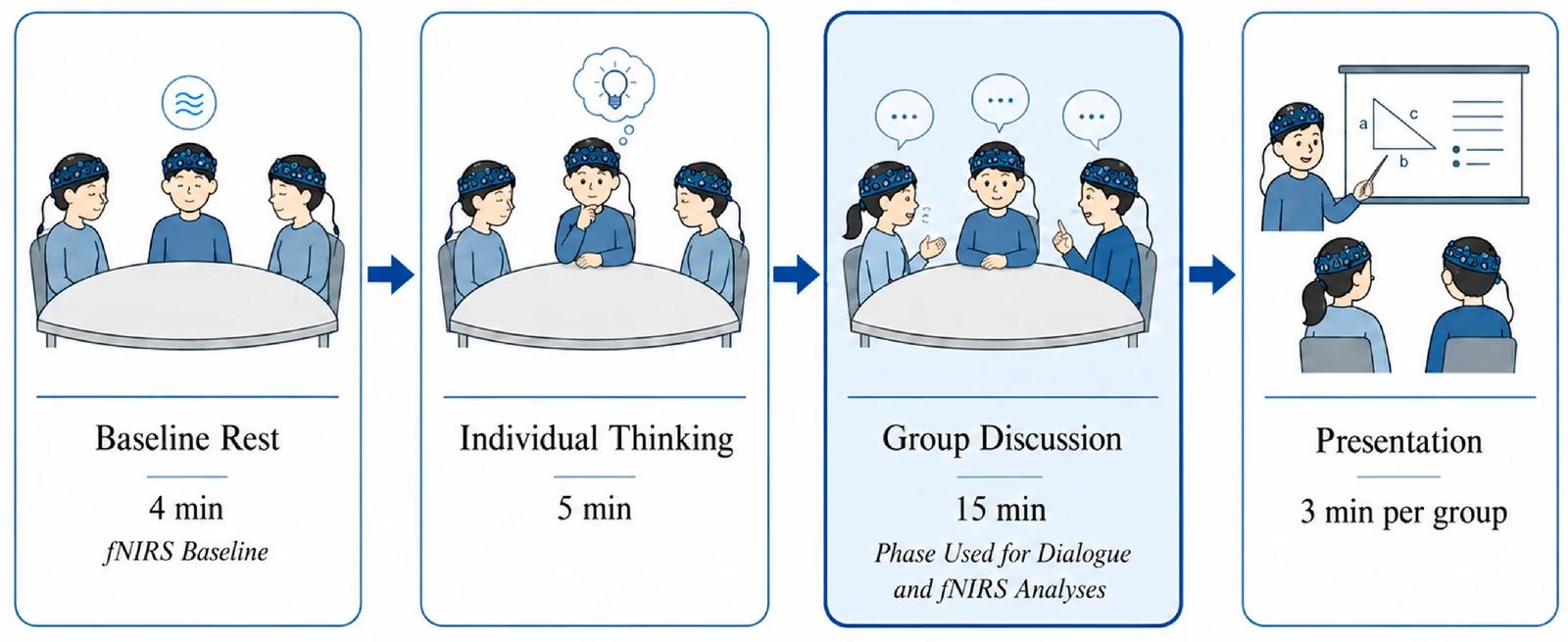
Procedure of the collaborative mathematical problem-solving task. Note. fNIRS = functional near-infrared spectroscopy.

To increase the ecological validity of the research context, students participated in classroom-like testing sessions. Each testing session included approximately 12 students, who were assigned to the pre-formed groups described above. The groups completed the two tasks in a fixed sequence, with the apartment spatial design task administered first and the campus sports event task administered second. Each task included four phases: a 4 min resting baseline, 5 min of individual thinking, 15 min of group discussion, and a 3 min group presentation. The procedure is shown in Figure 1. The group discussion phase was the primary phase used for accountable talk coding and fNIRS-based inter-brain synchrony analysis.

Three types of data were collected simultaneously during the tasks. First, individual speech was recorded using BOYA BY-V20 wireless microphones (Shenzhen Jiayz Photo Industrial Co., Ltd., Shenzhen, China), and the audio recordings were combined with video data to transcribe students’ dialogue and annotate turn-level timestamps. Second, group interaction was recorded using two vivo Y100 smartphones (vivo Mobile Communication Co., Ltd., Dongguan, China). Third, students’ brain activity was recorded using an Artinis Brite24 functional near-infrared spectroscopy (fNIRS) system (Artinis Medical Systems B.V., Elst, The Netherlands) at a sampling rate of 10 Hz. The final spatial design products completed by each group were collected and used to score task performance.

Because participants were children, child-adapted procedures were implemented during fNIRS recording. Cap sizes were selected according to participants’ head sizes, and optode placement was referenced to the international 10–20 system. Before formal recording, signal quality was assessed using OxySoft version 3.2.51.4 x64 (Artinis Medical Systems, Elst, The Netherlands), and adjustments to cap placement or optode–scalp contact were made when necessary. Task events were synchronized across acquisition computers using EventIDE experimental control software (version 2020-11-30), allowing fNIRS recordings from multiple participants to be aligned with the same task timeline. Participants were provided with a brief adaptation period before the collaborative task, and standardized instructions were provided.

### 2.3. Measures

The main variables, operational definitions, analytic levels, and corresponding research questions are summarized in Table 1.

#### 2.3.1. Accountable Talk Measures

All group discussions were transcribed into turn-by-turn text. Each conversational turn was annotated with a start time and an end time. The coding scheme was based on the accountable talk framework. In this study, accountable talk was operationalized as coded turns that reflected accountability to the learning community, accountability to knowledge, and accountability to reasoning. Accountability to the community mainly referred to responding to, accepting, repeating, or elaborating on peers’ contributions. Accountability to knowledge mainly referred to stating task facts, proposing design solutions, or evaluating the reasonableness of a solution. Accountability to reasoning mainly referred to explaining spatial relations, coordinating multiple constraints, or translating real-life needs into design criteria.

Accountable talk coding used the conversational turn as the basic unit of analysis. The coding was designed to identify task-relevant accountable discourse functions, including responding to peers, adding information, proposing design ideas, evaluating reasonableness, explaining spatial relations, or revising the design. The coding categories, operational definitions, and examples are provided in Supplementary Table S2.

Two researchers coded the transcripts using the coding manual. Before formal coding, the researchers were trained with sample transcripts to establish shared coding criteria. They then independently coded a subset of the transcripts, and interrater reliability was assessed using Cohen’s kappa. The agreement was acceptable, κ = 0.82. Disagreements were resolved through discussion, and the consensus codes were used in subsequent analyses.

At the group × task level, five accountable talk (AT) indicators were generated: the number of accountable talk turns, the total duration of accountable talk, the proportion of accountable talk duration relative to total speaking time, the mean duration of accountable talk turns, and accountable talk density. Accountable talk density was defined as the number of accountable talk turns per unit of student speaking time. At the temporal-window level, accountable talk density was further calculated for each window and aligned with the inter-brain synchrony measures from the same window for the temporal-window association analyses.

#### 2.3.2. Group Task Performance

Group task performance was scored based on the spatial design product completed by each group in each of the two tasks. The scoring unit was the final group-level spatial layout plan rather than individual student performance. Thus, each group × task record corresponded to one task performance score, yielding 30 task performance records.

The scoring rubric focused on five broad dimensions: completeness of spatial objects, reasonableness of spatial relations, satisfaction of quantitative constraints, clarity and verifiability of design representation, and coordination of multiple task constraints. Raters judged whether each group reasonably arranged the required functional areas, appropriately handled area, location, distance, circulation, and functional relations, provided necessary quantitative calculations and labels, and produced an overall feasible design. The full task-specific scoring rubric is provided in Supplementary Table S4.

Two raters independently scored the group products using the rubric. Before scoring, the raters were trained with sample products to establish shared criteria. Interrater agreement was calculated after scoring, and products with substantial discrepancies were discussed to determine the final score. To facilitate interpretation across models, task performance scores were standardized before being entered into the statistical models. The standardized score, labeled task_score_z, was used as the focal performance variable in RQ1, RQ2, and RQ3.

Interrater reliability was evaluated before resolving disagreements. The two raters showed excellent agreement for the group task-performance scores (two-way random-effects, absolute-agreement intraclass correlation coefficient, ICC(2, 1) = 0.99, 95% CI [0.97, 0.999]). The final consensus scores were used as the group task performance measure in subsequent analyses.

#### 2.3.3. fNIRS and Inter-Brain Synchrony Measures

Functional near-infrared spectroscopy hyperscanning was used to record group members’ brain activity simultaneously during the collaborative tasks. Because group-level IBS estimation can be influenced by motion artifacts, signal-quality variation, and systemic physiological fluctuations, preprocessing and quality-control procedures were implemented before IBS calculation. The fNIRS signal was sampled at 10 Hz. The main analysis focused on changes in oxygenated hemoglobin concentration, that is, the HbO signal. A 4 min resting baseline was recorded before each task and used for subsequent baseline correction.

The fNIRS preprocessing and inter-brain synchrony computation were performed in MATLAB R2021b (MathWorks, Natick, MA, USA). The raw fNIRS signal was first converted into optical density signals, and motion artifacts were corrected using a wavelet-based method. The signals were then band-pass filtered at 0.01–0.5 Hz and converted into HbO concentration changes. To reduce the influence of global shared components, including potential non-neural physiological contributions, principal component analysis (PCA) regression was applied before IBS estimation. The cumulative explained variance threshold was predefined at 0.75 to balance the reduction in shared variance and the retention of task-related signal. This threshold was selected prior to statistical analyses and was not optimized based on the observed IBS results. The preprocessed HbO time series were baseline-corrected before inter-brain synchrony calculation.

Channel-level quality control was applied to the fNIRS data. At the record level, a threshold was set based on the proportion of low-quality channels. If the proportion of low-quality channels in a group × task record was equal to or greater than one-sixth of all channels, the record was excluded from subsequent inter-brain synchrony analyses. If the proportion of low-quality channels was below one sixth, the record was retained. For retained records, low-quality channels were excluded at the channel level and were not included in wavelet transform coherence calculations or channel-group averages. Channel-group-level inter-brain synchrony was calculated using the remaining valid channels within each channel group. Adjacent-channel substitution was not used in the main analysis.

Inter-brain synchrony (IBS) was estimated using wavelet transform coherence (WTC). We calculated coherence values between homologous-channel HbO time series for group members in the task-relevant low-frequency band of 0.02–0.08 Hz. For each group, channel-level coherence values were first calculated for all member pairs and then averaged within the group to generate group-level IBS indicators.

**Figure 2:**
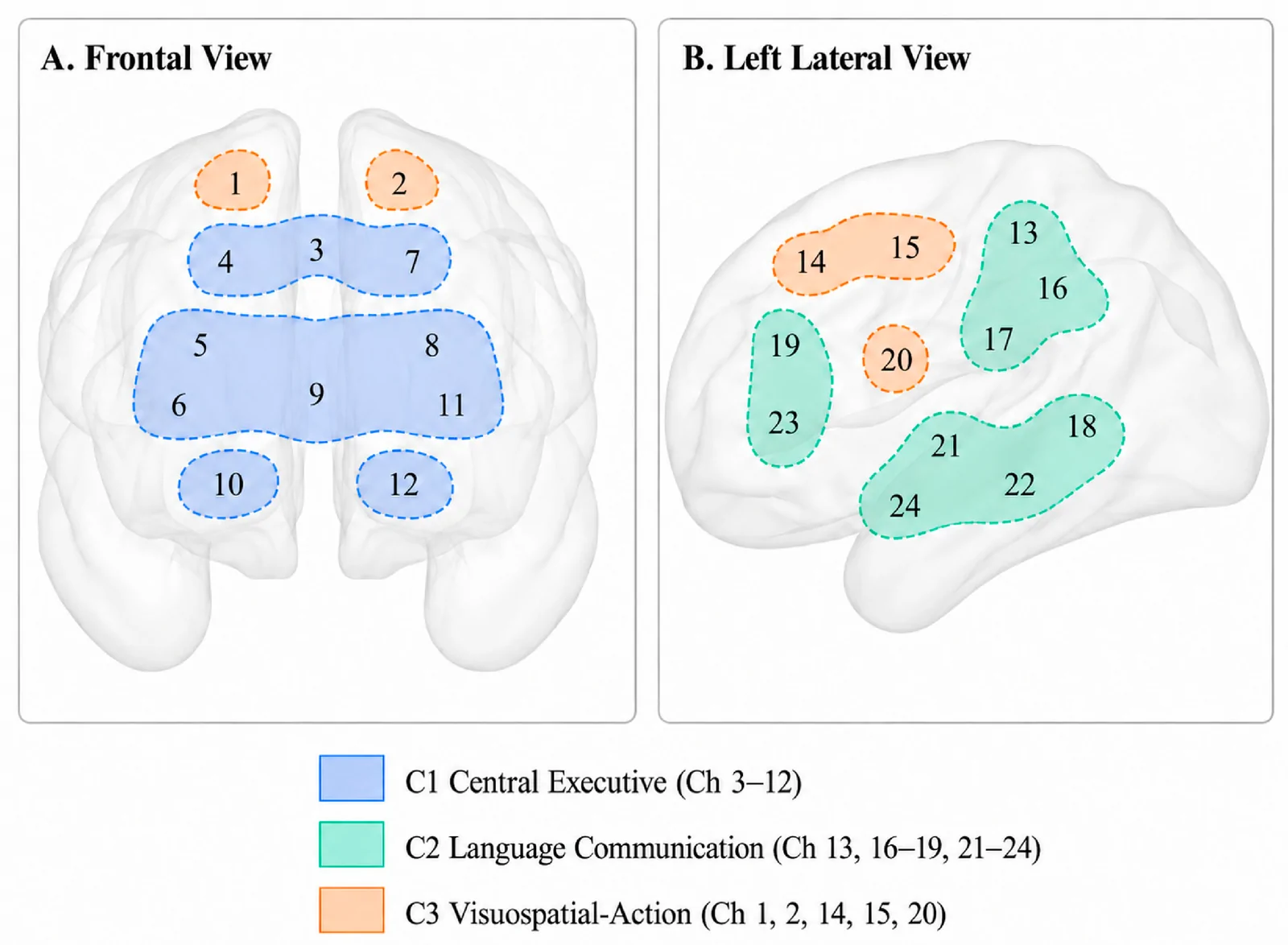
fNIRS channel layout and analytical channel group assignment. Note. Shaded areas indicate the approximate spatial coverage of channels within each channel group. C1 = executive-function-related channel group, Channels 3–12; C2 = language-related channel group, Channels 13, 16–19, and 21–24; C3 = visuospatial/action-related channel group, Channels 1, 2, 14, 15, and 20. The shaded areas represent channel-level functional grouping rather than strict anatomical boundaries. fNIRS = functional near-infrared spectroscopy; Ch = channel.

Based on channel location and task function, the fNIRS channels were assigned to three channel groups: C1, the executive-function-related channel group; C2, the language-related channel group; and C3, the visuospatial/action-related channel group. The channel layout and channel-group mapping are shown in Figure 2. The channel grouping is reported in Table 2, and the channel coordinates and anatomical labels are provided in Supplementary Table S3. IBS indicators were generated at two levels: group × task × fNIRS channel group and temporal window × fNIRS channel group. These indicators were used for the channel-group-specific analyses and the temporal-window association analyses.

### 2.4. Temporal Alignment and Window-Level Data Construction

The discourse data and fNIRS data were aligned to the same task timeline. First, video transcripts, turn-level timestamps, and fNIRS time series were synchronized according to task onset. Each conversational turn had a start time and an end time, and the fNIRS data were segmented using the same timeline. Segments that may have been affected by task onset, device adjustment, or task termination were removed. Only valid collaborative discussion segments that passed synchronization and quality checks were retained.

After temporal synchronization, sliding windows were used to characterize the temporal dynamics of collaborative activity and inter-brain synchrony. The window length was 240 s, and the step size was 20 s. Within each window, accountable talk density and IBS in each channel group were calculated. Accountable talk density was calculated based on the number of accountable talk turns and the amount of student speaking time within the window. IBS was first estimated at the channel level and then aggregated to the three channel groups: C1, the executive-function-related channel group; C2, the language-related channel group; and C3, the visuospatial/action-related channel group.

The final window-level dataset contained 900 valid temporal windows. After each window was expanded across the three channel groups, the dataset contained 2700 temporal window × fNIRS channel group records. These records were treated as repeated measurements of within-task temporal variation and inter-brain synchrony patterns rather than independent observations. To analyze temporal changes in accountable talk density, relative task time was calculated for each sliding window at the group × task level. It represented the normalized temporal position of each window within the valid collaborative discussion segment and reflected the progression of the group-level collaborative process rather than individual students’ speaking duration or participation time.

### 2.5. Statistical Analysis

The analyses in this study focused on collaborative group activity rather than individual-level inference. Statistical analyses were conducted in R version 4.5.3 (R Foundation for Statistical Computing, Vienna, Austria). The 15 collaborative groups were the independent sampling units, yielding 30 repeated group × task observations. These measurements were treated as repeated observations rather than additional independent sampling units. Mixed-effects models were used when the model structure allowed reliable estimation of random effects. When a candidate mixed-effects model showed singularity, the corresponding analysis was simplified to a fixed-effects model, and this decision was explicitly reported. Continuous predictors were z-standardized before being entered into the models. Dependent variables were retained on their original scales unless otherwise specified. False discovery rate (FDR) correction was applied for multiple comparisons, and all statistical tests were two-sided. The statistical analyses were organized according to the hierarchical structure of the data and the corresponding research questions. Specifically, RQ1 examined associations between collaborative task performance and accountable talk characteristics at the group × task level; RQ2 examined inter-brain synchrony patterns across channel groups and task conditions; and RQ3 examined the within-window association between accountable talk density and IBS at the temporal-window level.

For RQ1, analyses were conducted at the group × task level to examine the association between task performance and accountable talk characteristics. The five accountable talk indicators were modeled separately as dependent variables. The focal predictor was standardized task performance, task_score_z. Task and group size were included as covariates. A supplementary temporal-window analysis used accountable talk density as the dependent variable and compared a linear time model, a task performance × time interaction model, and a natural spline time model.

For RQ2, channel group means and contrasts were obtained from the window-level model controlling for accountable talk density, task performance, task, group size, and total student speaking time, with a group random intercept. To examine whether performance was differentially associated with IBS across channel groups, we further aggregated IBS estimates to the group × task × channel group level and conducted the performance-by-channel-group analysis using 90 observations derived from the 30 repeated group × task units. For this aggregated performance-by-channel-group model, the IBS outcome was z-standardized before model fitting. The final linear regression included the performance × channel group interaction. If the interaction was significant, simple slopes were estimated within each channel group and adjusted using FDR correction. Task-specific channel group models were conducted as exploratory supplementary analyses.

RQ3 used temporal-window and channel-group measurements as repeated observations nested within collaborative group × task episodes to characterize the dynamic association between accountable talk density and IBS. Window-level IBS was modeled as the dependent variable. Standardized accountable talk density was entered as the focal predictor, with task, group size, total student speaking time, and fNIRS channel group included as covariates. The AT_density_z × task_score_z interaction was then added to the model. If the interaction was significant, simple slopes were estimated at task_score_z = −1, 0, and +1.

Because the sliding-window data involved overlapping temporal observations, window-level measurements were treated as repeated observations within collaborative episodes rather than independent samples. Therefore, window-level findings were interpreted as temporal associations within observed collaborative episodes rather than as estimates derived from treating overlapping windows as independent observations. To examine whether the observed patterns depended on temporal overlap, a sensitivity analysis using non-overlapping 240 s windows was conducted. This analysis retained the same IBS estimation procedure, channel-group definitions, and behavioral indicators while reducing temporal overlap between adjacent observations. The results of this sensitivity analysis are reported in Section 3.

## 3. Results

### 3.1. Associations Between Task Performance and Accountable Talk Characteristics

RQ1 examined whether higher-performing groups showed different accountable talk characteristics. The unit of analysis was the group × task record, with 30 records in total. Five accountable talk indicators were modeled separately as dependent variables: the number of accountable talk turns, the total duration of accountable talk, the proportion of accountable talk duration relative to total speaking time, the mean duration of accountable talk turns, and accountable talk density. Standardized task performance was entered as the focal predictor. The models controlled for task and group size and included a random intercept for group. The main model results are summarized in Table 3.

Task performance was significantly and positively associated with the number of accountable talk turns. For each one-standard-deviation increase in task performance, the number of accountable talk turns increased by 19.889 turns on average, *p* = 0.005, FDR-adjusted *p* = 0.014. Task performance was not significantly associated with the total duration of accountable talk, estimate = 0.773, *p* = 0.979. The proportion of accountable talk duration showed a positive trend, estimate = 0.042, *p* = 0.089, but did not reach statistical significance after FDR correction, FDR-adjusted *p* = 0.112.

Further results showed that task performance was significantly and negatively associated with the mean duration of accountable talk turns, estimate = −0.472, *p* = 0.022, FDR-adjusted *p* = 0.036. Task performance was also significantly and positively associated with accountable talk density, estimate = 2.699, *p* < 0.001, FDR-adjusted *p* < 0.001. These results indicate that higher-performing groups were characterized by more numerous, denser, and shorter accountable talk turns, rather than by a longer total duration of accountable talk.

**Figure 3:**
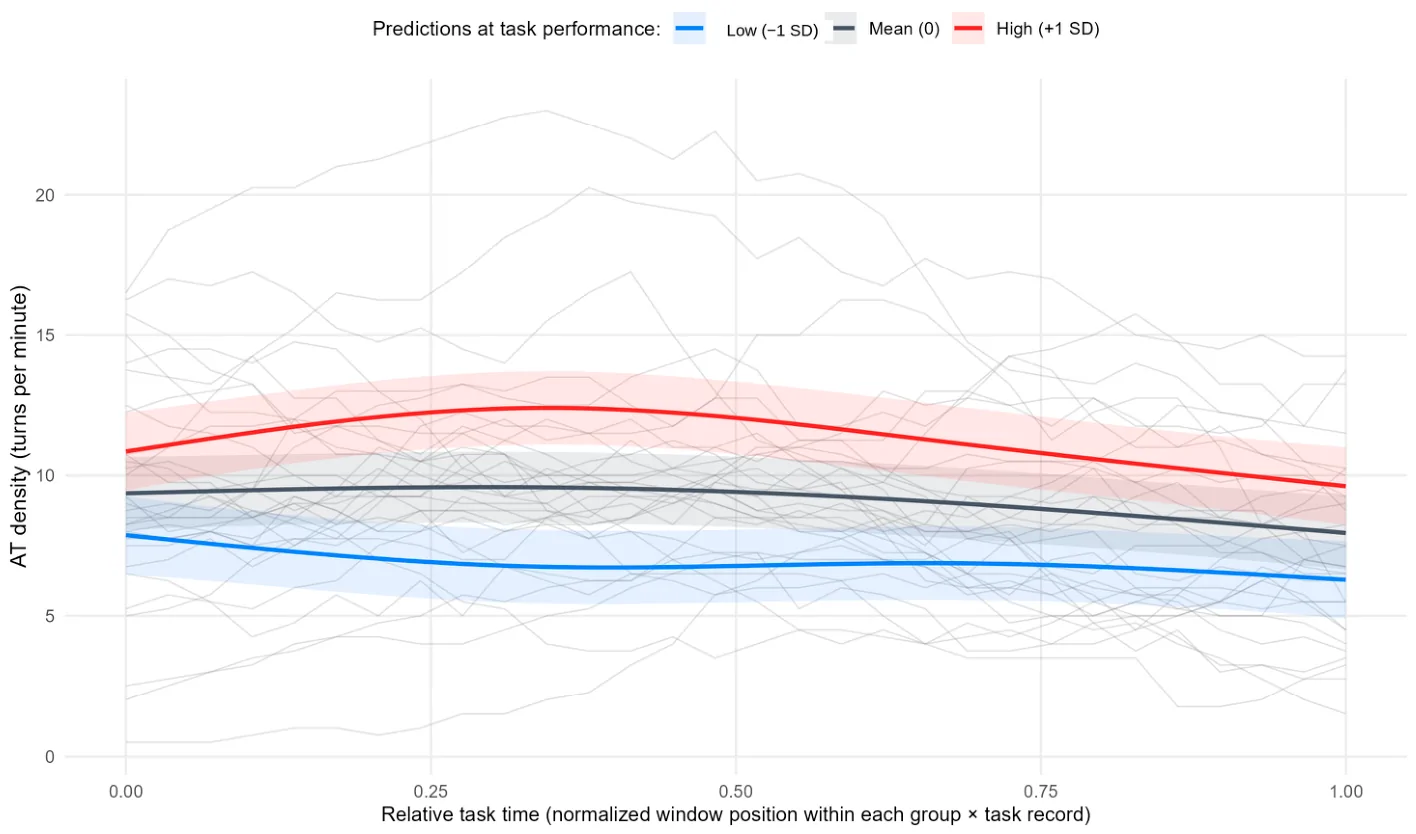
Temporal trajectory of AT density by task performance. Note. Thin light-gray lines represent observed AT density trajectories for individual group × task records. The blue, black, and red smooth lines show natural spline-based model-predicted trajectories at lower (−1 *SD*), mean (0 *SD*), and higher (+1 *SD*) task performance levels, with shaded bands representing 95% model-based confidence intervals. Relative task time denotes the normalized position of each 240 s sliding window within the valid collaborative discussion segment. Adjacent windows were advanced by 20 s and therefore partially overlapped. AT = accountable talk; *SD* = standard deviation.

A supplementary temporal-window analysis further examined the distribution of accountable talk density across the task. The baseline model showed that higher-performing groups had higher accountable talk density at the temporal-window level, b = 2.229, *p* < 0.001. The main effect of time was negative, b = −0.436, *p* < 0.001. After adding the task performance × linear time interaction, model fit showed a trend toward improvement, *p* = 0.051. When a natural spline function was used to model nonlinear temporal trajectories, model fit improved significantly, χ^2^(5) = 47.89, *p* < 0.001. The predicted temporal trajectories of accountable talk density are shown in Figure 3.

Overall, the RQ1 results showed that higher-performing groups did not produce longer accountable talk. Instead, they produced accountable talk more frequently and more compactly during the task.

### 3.2. Neural Synchrony Patterns Associated with Collaborative Performance

Building on the behavioral interaction patterns identified in RQ1, RQ2 examined whether collaborative performance was also associated with distinct patterns of inter-brain synchrony. We first estimated channel-group-level synchrony patterns using sliding-window IBS measurements and then aggregated these estimates at the group × task level for performance-related comparisons. The analysis incorporated repeated window-level measurements when estimating channel-group-level synchrony patterns.

**Figure 4:**
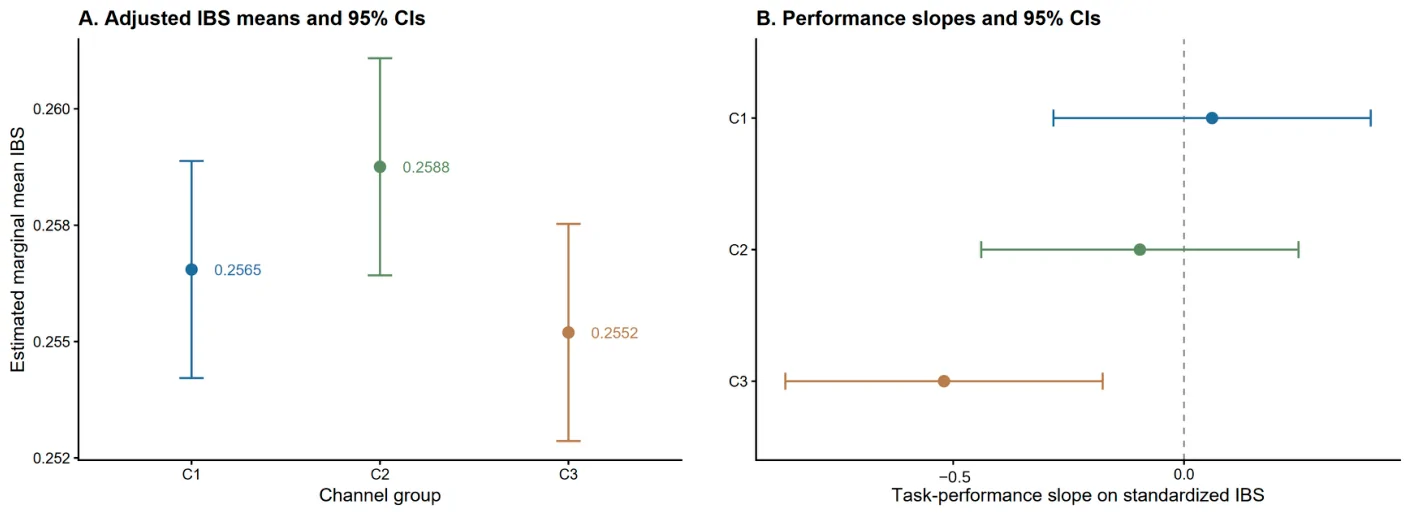
Channel-group differences in inter-brain synchrony. Note. Panel (**A**) shows the estimated marginal means of inter-brain synchrony (IBS) across the three channel groups, with error bars representing 95% confidence intervals derived from the statistical model. Panel (**B**) shows simple slopes for the association between standardized task performance and IBS within each channel group, with horizontal bars representing 95% confidence intervals and the vertical dotted line indicating zero. C1 = central executive channel group; C2 = language-related channel group; C3 = visuospatial/action-related channel group; IBS = inter-brain synchrony.

The results showed that task performance was associated with differential IBS patterns across channel groups. The performance × channel group interaction was significant (likelihood ratio test: χ^2^(2) = 6.52, *p* = 0.038), indicating that the association between task performance and IBS differed across channel groups. FDR-adjusted simple slopes were subsequently examined within each channel group. This finding suggests that the association between collaborative performance and neural synchrony differed across channel groups during collaborative problem solving. The adjusted channel-group means and corresponding performance slopes are summarized in Figure 4.

To examine whether this pattern was influenced by the temporal overlap of adjacent sliding windows, we conducted a sensitivity analysis using non-overlapping 240 s windows. The direction of the performance-by-channel-group association remained consistent (β = −0.491, *p* = 0.050), although the statistical evidence was weaker under the more conservative sampling strategy (χ^2^(2) = 4.31, *p* = 0.116). Thus, the direction of the channel-group-related association was preserved, although the estimate became less precise under the more conservative temporal sampling strategy. Sensitivity analyses across PCA explained-variance thresholds of 50%, 60%, and 75% showed that the overall performance × channel-group interaction remained directionally consistent across PCA specifications, although the strength of statistical evidence varied after multiple-comparison correction (Supplementary Table S7).

### 3.3. Window-Level Association Between Accountable Talk and Neural Synchrony

Integrating the behavioral interaction patterns identified in RQ1 and the neural synchrony patterns examined in RQ2, RQ3 investigated whether accountable talk density and IBS showed a within-window association during collaborative activity. Rather than treating discourse behaviors as static group characteristics, this analysis focused on the association between temporal variation in accountable talk density and inter-brain synchrony.

**Figure 5:**
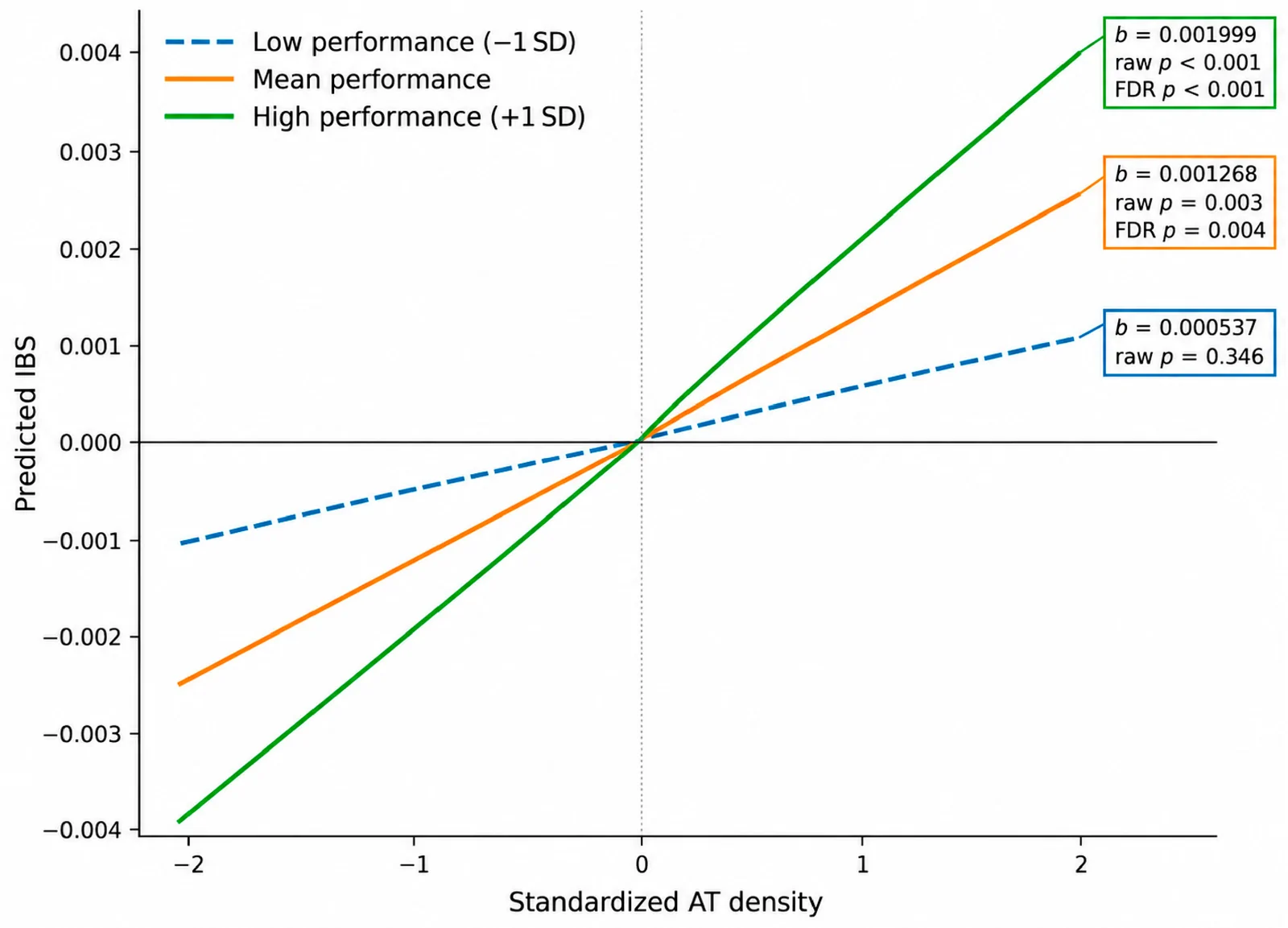
Performance-dependent association between AT density and inter-brain synchrony. Note. Lines represent model-implied simple slopes for the association between standardized AT density and predicted inter-brain synchrony at lower task performance (−1 *SD*), mean task performance, and higher task performance (+1 *SD*). Raw *p*-values are shown for all simple slopes; FDR-adjusted *p*-values are additionally reported for slopes that remained significant after correction. AT = accountable talk; IBS = inter-brain synchrony; *SD* = standard deviation; FDR = false discovery rate.

The results showed that AT density was associated with IBS during collaborative problem solving. In the original overlapping-window analysis, AT density was positively related to IBS (b = 0.001783, *p* < 0.001). Furthermore, the association between AT density and IBS was moderated by task performance, as indicated by a significant AT density × task performance interaction (b = 0.000731, *p* = 0.004), with the corresponding performance-dependent simple slopes shown in Figure 5. These estimates represent temporal associations within collaborative episodes based on repeated window-level measurements.

We further examined whether these associations were sensitive to temporal sampling strategy using non-overlapping 240 s windows and aggregated analyses. The AT-related estimates remained directionally positive but became less precise under the more conservative temporal sampling approach. After aggregation to the group × task × channel-group level, the AT main effect was not significant (*p* = 0.230), whereas the AT density × task performance interaction remained significant in the primary 75% PCA specification (*p* < 0.001). Additional sensitivity analyses using alternative PCA explained-variance thresholds (50%, 60%, and 75%) showed generally consistent directional patterns for the RQ3 associations, although the statistical significance of the AT density × task performance interaction varied across PCA specifications (Supplementary Tables S6 and S7). These results suggest that the association between accountable talk and IBS may depend on collaborative performance conditions rather than reflecting a uniform association across groups and tasks.

## 4. Discussion

This study examined the relations among accountable talk, inter-brain synchrony (IBS), and task performance in elementary students’ collaborative spatial design tasks. These findings can be understood from three complementary levels of collaborative processes: how groups organize task-relevant interaction through accountable talk, how collaborative activity is associated with channel-group-specific inter-brain synchrony patterns, and how behavioral and neural measures showed temporally varying associations during collaborative activity. Taken together, the findings suggest that group task performance cannot be represented simply by more talk or by higher overall neural synchrony. Higher-performing groups were primarily characterized by denser accountable talk within limited speaking time. IBS showed channel-group-specific patterns, and the association between accountable talk density and IBS depended on group task performance. Thus, the central finding is not a general increase in either a behavioral or neural indicator, but a performance-dependent association between task-relevant accountable discourse and neural synchrony during collaborative problem solving.

### 4.1. Dense Accountable Talk as Conversational Iteration

The RQ1 findings showed that higher-performing groups did not spend more time on accountable talk. Instead, they produced more numerous, shorter, and denser accountable talk turns within limited speaking time. This highlights that, in the present collaborative tasks, accountable talk may be better understood by considering how task-relevant turns are organized rather than only whether such talk occurs. If a group relies mainly on a few long explanations from one or two students, while other members provide little timely response, evaluation, or revision, the talk may not become shared reasoning. In contrast, dense and short accountable talk turns may reflect more frequent opportunities for task-relevant exchange and coordination during collaborative work.

This interpretation is consistent with the basic orientation of accountable talk. Michaels et al. (2008) emphasized that the value of classroom discourse lies not merely in speaking, but in whether students make their contributions accountable to the learning community, to knowledge, and to standards of reasoning. The present findings extend this view by suggesting that, in small-group collaborative problem solving, the occurrence of accountable talk may also be associated with how these task-relevant turns are temporally distributed during collaboration. Similarly, de Araujo et al. (2025) argued that ideas become group resources only when peers acknowledge, respond to, and develop them. In this sense, accountable talk density may provide a more informative indicator of task-relevant interaction than total accountable talk duration in this context.

The temporal-window results further showed that the advantage of higher-performing groups in accountable talk density was concentrated mainly in the early and middle phases of the task, rather than being constant throughout the discussion. This pattern is consistent with the process structure of open-ended spatial design. During the early and middle phases, students need to understand the situation, clarify constraints, generate possible solutions, and coordinate competing requirements. Once a design becomes more stable, later phases may shift toward drawing, execution, and preparation for presentation. Yan et al. (2025) emphasized that multimodal research on collaborative learning should attend to temporal organization rather than relying only on whole-task averages. These findings suggest that accountable talk density should be interpreted not only in terms of its overall level but also in relation to its temporal distribution across the task.

### 4.2. Inter-Brain Synchrony Patterns Across Channel Groups

Building on the behavioral interaction patterns identified in RQ1, the RQ2 findings examined whether these differences in collaborative processes were accompanied by distinct patterns of inter-brain synchrony. The results indicated that IBS varied across channel groups during collaborative spatial design, although the absolute differences among channel groups were small. The C2 language-related channel group showed the highest estimated synchrony, whereas task performance was negatively associated with synchrony in the C3 visuospatial/action-related channel group. Pairwise contrasts further showed that these differences were statistically reliable but limited in magnitude (maximum difference = 0.00356 IBS units). Therefore, these findings should be interpreted as subtle variations in channel-group-level IBS rather than evidence of strong functional separation among cortical systems. This pattern suggests that IBS should not be interpreted as a global positive indicator of collaborative activity. Réveillé et al. (2024) similarly argued that IBS in team and collaborative settings needs to be interpreted in relation to task demands and interactional states, rather than being treated as a direct marker of better collaboration.

The higher synchrony observed in C2 is consistent with the possibility that language-related processes contributed to task-relevant coordination, although IBS alone cannot identify the underlying cognitive mechanism. Although the tasks required students to produce spatial layouts and drawings, group members needed to use language to interpret task conditions, describe spatial relations, compare design options, and coordinate area, location, function, and circulation constraints. In this sense, language provided an important medium through which individual ideas could be transformed into a shared design plan. De Felice et al. (2025) suggested that synchrony in language-related regions may be associated with processes involved in interpersonal communication and shared task coordination. The higher C2 synchrony observed in the present study is consistent with the possibility that language-related neural synchrony was involved in task-relevant collaborative interaction, although the present data cannot determine the specific cognitive processes underlying this association.

The negative association between C3 synchrony and task performance requires more cautious interpretation. This result does not mean that lower synchrony is generally better. Rather, it suggests that, in this spatial design task, higher synchrony in the visuospatial/action-related channel group may have different process meanings. Higher C3 synchrony may be related to shared engagement with visuospatial aspects of the task, but it may also indicate that group members remained jointly engaged in layout adjustment, drawing operations, or unresolved spatial difficulty. Recent child–child fNIRS hyperscanning research in a naturalistic Tangram task similarly found that inter-brain coupling was not determined simply by the presence of collaboration, while exploratory evidence suggested that coupling may covary with task-related manual engagement (da Silva Soares Junior & Sato, 2026). Zhang et al. (2024) also found that higher IBS did not necessarily correspond to better behavioral performance in collaborative problem solving; different collaboration modes may produce different neural synchrony patterns. Therefore, the negative C3 association in the present study is better interpreted as a task-dependent and channel-group-specific pattern, rather than as evidence that lower synchrony is generally beneficial. Because the present study did not align C3 synchrony frame by frame with drawing behavior, hand movements, gaze allocation, or spatial reasoning talk, this interpretation remains preliminary.

### 4.3. Performance-Dependent Behavioral–Neural Association

Integrating the behavioral interaction patterns identified in RQ1 and the neural synchrony patterns examined in RQ2, RQ3 further investigated whether accountable talk density and IBS showed a temporal association during collaborative activity. The findings showed that the relationship between accountable talk density and IBS varied across task performance levels. At the temporal-window level, accountable talk density was associated with IBS, but this association was moderated by task performance. Given the repeated temporal structure of overlapping windows, these findings should be interpreted as temporal associations within the analyzed collaborative episodes.

These findings are consistent with a process-oriented view of inter-brain coordination. Dynamic hyperscanning research has shown that inter-brain network states fluctuate over time and are associated with specific communication behaviors and group performance (Wang et al., 2022). More broadly, second-person neuroscience situates cross-brain synchrony within ongoing social interaction, communication, coordination, and learning (Schilbach & Redcay, 2025). The present study extends this perspective by temporally aligning task-relevant accountable discourse with IBS within the same collaborative episodes, thereby linking an observable discourse process to moment-to-moment variation in neural coordination.

The robustness analysis at the aggregated level further constrained this interpretation. When the data were aggregated to the group × task × channel group level, the main effect of accountable talk density was no longer significant, whereas the interaction between accountable talk density and task performance remained significant. Thus, the findings do not indicate a general association between denser accountable talk and higher average IBS across all groups; rather, the discourse–neural relationship varied with collaborative performance.

### 4.4. Implications for Multimodal Collaboration Research and Classroom Support

Taken together, the three levels of evidence identified in this study suggest that collaborative processes in the present tasks may involve coordinated patterns of interaction organization, inter-brain synchrony, and their temporal associations. Rather than reflecting a simple increase in any single measure, successful collaboration was associated with the organization of task-relevant discourse and performance-related variation in channel-group-specific inter-brain synchrony. Cukurova et al. (2020) argued that multimodal learning analytics becomes more interpretable when theoretical constructs, data sources, and analytic units are aligned. The present study illustrates the value of examining discourse, task performance, and fNIRS measures within the same task-based temporal structure.

For classroom support, the findings do not suggest that teachers should simply encourage longer discussions, higher speaking frequency, or stronger synchrony. A more direct implication is that teachers may need to attend to whether students engage in task-relevant exchanges involving proposing, responding, evaluating, and revising. Kollar et al. (2006) showed that collaboration scripts can support more effective collaborative processes by structuring interaction roles and discourse sequences. Consistent with this view, accountable talk scaffolds may be most useful when they help students create interactional conditions in which individual ideas can be shared, examined, and revised by group members, rather than when they merely make group discussion more active.

### 4.5. Limitations and Future Directions

Several limitations should be acknowledged. First, the independent sampling units were the 15 collaborative groups, which limits the precision and generalizability of the estimated effects. The 95% confidence intervals in Supplementary Table S5 describe estimation uncertainty conditional on the fitted models, but they do not replace validation with larger independent samples. The interaction estimates, simple slopes, and channel-group-specific findings should therefore be interpreted as associations observed in this sample and require confirmation with more independent groups. In addition, the study used an associational design. The temporal-window analyses showed that accountable talk density and IBS were associated within the analyzed temporal framework, but they cannot establish that accountable talk caused neural synchrony, or that neural synchrony directly improved task performance. Future studies should test these patterns with larger group-level samples across multiple classrooms and task contexts. Experimental manipulation of accountable talk scaffolds, task interdependence, or role assignment would also help clarify possible causal pathways.

Future research could extend this paradigm to adolescent samples to examine whether the observed associations among accountable talk, inter-brain synchrony, and collaborative task performance generalize across developmental stages and whether developmental changes in peer interaction influence behavioral–neural association during collaboration.

Second, the two collaborative tasks were administered in a fixed sequence, with the apartment task always preceding the sports-event task. Although the tasks were designed to share comparable structural characteristics and cognitive demands, task identity was therefore fully confounded with administration order. Consequently, the present study cannot distinguish task-specific effects from possible practice, familiarity, fatigue, or other order-related effects. Future studies should counterbalance or randomize task order to separate these sources of variation more clearly.

Third, the interpretation of channel-group-specific IBS requires caution. The C1, C2, and C3 channel groups were defined based on channel locations, sampled cortical regions, and task-relevant functions, rather than as strict anatomical regions of interest. In particular, the negative association between C3 synchrony and task performance cannot yet be interpreted as evidence that higher-performing groups had completed spatial understanding or achieved more efficient division of labor. Future research should align C3 synchrony more closely with drawing behavior, hand movements, gaze allocation, and spatial reasoning discourse to clarify its process-level meaning.

Fourth, temporal-window multimodal alignment requires further methodological development. Different window lengths, lag structures, and approaches for modeling temporal dependence may influence the estimation and interpretation of behavioral–neural associations. Although the non-overlapping window sensitivity analysis showed that the direction of the observed associations was generally preserved, some effects became less precise under the more conservative temporal sampling strategy. Future studies should further examine temporal dependence using alternative lagged or time-series approaches. Therefore, the present within-window behavioral–neural associations should be interpreted as performance-dependent behavioral–neural associations within the analyzed temporal framework rather than as effects that are invariant across different sampling strategies.

## 5. Conclusions

This study examined how accountable talk, inter-brain synchrony, and task performance were related during elementary students’ collaborative spatial design tasks. The findings suggest that group task performance was not equivalent to more talk or higher overall neural synchrony. Higher-performing groups were more clearly characterized by dense and temporally organized accountable talk, while the IBS–performance associations varied across channel groups. More importantly, the temporal association between accountable talk density and IBS was moderated by task performance. These findings provide preliminary multimodal evidence that collaborative activity may involve performance-dependent temporal associations between behavioral interaction and inter-brain synchrony, rather than a static advantage in any single behavioral or neural indicator.

## Figures and Tables

**Table 1:** Measures, analytic levels, and research questions.

Measure	Operational Definition	Analytic Level	Used In
AT count	Number of accountable talk turns.	Group × task	RQ1
AT duration	Total duration of accountable talk turns.	Group × task	RQ1
AT duration proportion	Proportion of total speaking time coded as accountable talk.	Group × task	RQ1
Mean AT turn duration	AT duration divided by AT count.	Group × task	RQ1
AT density	Number of accountable talk turns per unit of student speaking time.	Group × task; time window	RQ1, RQ3
Task score	Scored quality of the group design product.	Group × task	RQ1–RQ3
IBS	WTC-based inter-brain synchrony.	Group × task × channel group; time window × channel group	RQ2, RQ3
Relative task time	Normalized position of each time window within the group discussion phase.	Time window	RQ1

Note. AT = accountable talk; IBS = inter-brain synchrony; WTC = wavelet transform coherence. “Group × task” refers to one group completing one collaborative spatial design task. “Time window × channel-group” refers to one time window expanded across the three channel groups used for IBS analysis.

**Table 2:** Assignment of fNIRS channels to analytical channel groups.

Channel Group	Channels	Main Channel Locations	Task-Related Interpretation
C1: Central Executive	3–12	Superior frontal and frontopolar regions, including BA9/BA10-related channels	Executive control, working memory, strategic planning, and task monitoring
C2: Language Communication	13, 16–19, 21–24	Inferior frontal, supramarginal, and temporal regions, including BA21/22/39/40/44-related channels	Speech production, verbal expression, auditory comprehension, semantic processing, and verbal coordination
C3: Visuospatial-Action	1, 2, 14, 15, 20	Superior/medial frontal, precentral, and postcentral regions, including BA6/BA8/BA9-related and sensorimotor channels	Visual attention, visuospatial processing, sensorimotor coordination, and drawing-related action

Note. BA = Brodmann area; fNIRS = functional near-infrared spectroscopy. Channel locations are based on fNIRS channel registration and anatomical labeling. Analytical channel groups represent task-relevant cortical regions sampled by fNIRS channels and do not correspond to canonical channel groups.

**Table 3:** Summary of primary statistical model results.

RQ	Outcome/Model	Key Effect	Estimate/Statistic	Inference
RQ1	AT turn count	Task performance	19.889	raw *p* = 0.005; FDR-adjusted *p* = 0.014; group × task
RQ1	Mean AT turn duration	Task performance	−0.472	raw *p* = 0.022; FDR-adjusted *p* = 0.036; group × task
RQ1	AT density	Task performance	2.699	raw *p* < 0.001; FDR-adjusted *p* < 0.001; group × task
RQ1	AT density trajectory	Natural spline model improvement	χ^2^(5) = 47.89	raw *p* < 0.001; group × task temporal trajectory
RQ2	IBS	Channel group contrast: C2 vs. C3	0.0036	raw *p* < 0.001; FDR-adjusted *p* < 0.001; group × task × channel group
RQ2	IBS	Task performance × channel group	χ^2^(2) = 6.52	raw *p* = 0.038; group × task × channel group
RQ2	C3 IBS	Task performance simple slope	−0.520	raw *p* = 0.003; FDR-adjusted *p* = 0.010; group × task × channel group
RQ3	IBS	AT density	0.00178	raw *p* < 0.001; FDR-adjusted *p* < 0.001; window × channel group
RQ3	IBS	AT density × task performance	0.000731	raw *p* = 0.004; FDR-adjusted *p* = 0.006; window × channel group
RQ3	Mean IBS	AT density × task performance	0.001750	raw *p* < 0.001; FDR-adjusted *p* < 0.001; aggregated group × task × channel group

Note. AT = accountable talk; IBS = inter-brain synchrony; FDR = false discovery rate. Values labeled as *p* are raw *p*-values; FDR-adjusted *p*-values refer to Benjamini–Hochberg-adjusted *p*-values. Estimates are reported for focal fixed effects; omnibus likelihood-ratio tests are reported as χ^2^ statistics. Selected model results and their 95% confidence intervals, including simple slopes and aggregated robustness results, are reported in Supplementary Table S5. Additional model outputs are retained in the analysis records.

## Data Availability

The data presented in this study are not publicly available due to privacy and ethical restrictions involving minors, classroom video/audio recordings, and fNIRS recordings. Student-identifiable images or videos are not publicly shared. De-identified derived data and analysis code may be made available from the corresponding author upon reasonable request, subject to institutional ethics approval.

## Supplementary Materials

The following supporting information can be downloaded at: https://www.mdpi.com/article/10.3390/bs16101787/s1, Supplementary Text S1: Representative collaborative spatial design task: Apartment spatial design; Supplementary Figure S1: Original spatial boundary diagram for the apartment spatial design task; Supplementary Table S1: Structural equivalence and baseline performance of the parallel collaborative tasks; Supplementary Table S2: Operational definitions, decision rules, and examples of accountable talk codes; Supplementary Table S3: fNIRS channel mapping, preprocessing, and quality control; Supplementary Table S4: Scoring framework for the apartment spatial design task; Supplementary Table S5: Full model results with 95% confidence intervals; Supplementary Table S6: PCA Regression Sensitivity Summary Across Explained-Variance Thresholds; Supplementary Table S7: Sensitivity Analyses of IBS Findings Across PCA Explained-Variance Thresholds.
