# Supplementary material for: Neural-Discursive Dynamics in Collaborative Spatial Problem Solving: Evidence from fNIRS Hyperscanning and Accountable Talk
Source: Behav Sci (Basel). 2026 Oct 1;16(10):1787. doi: 10.3390/bs16101787 (PMC13701405; doi:10.3390/bs16101787)
Supplement: Supplementary file 1 [file behavsci-16-01787-s001.zip › behavsci-4456482-supplementary.pdf]

Supplementary Materials

Supplementary Text S1. *Representative Collaborative Spatial Design Task: Apartment Spatial Design*

This study used two parallel collaborative spatial design tasks. To illustrate the structure of the task while preserving the usability of the remaining task version for future research, one representative task is provided below. The structural comparability of the two task versions is summarized in Supplementary Table S1.

The task was designed as an ill-structured, mathematics-rich spatial design problem. Rather than producing a single predetermined answer, students were required to translate contextual requirements into quantitative and spatial constraints, generate a feasible floor-plan solution, and represent their design using dimensions and necessary calculations. The task therefore provided opportunities for students to engage in mathematical modeling and elementary engineering-design-oriented practices, including constraint coordination, spatial representation, and design justification.

Note: The task was administered in Chinese and translated into English for review.

English Translation of the Task Text

Xiao Ming's family is planning to move from their current 60 m^2 two-bedroom apartment to a new 80 m^2 three-bedroom apartment. The locations of the entrance door and windows in the new apartment are fixed, as shown in Supplementary Figure S1. Please design and draw the floor plan for the new apartment according to the following requirements, and write down any necessary calculation steps in the calculation area.

Design Requirements

1. The new apartment should include at least a master bedroom, a secondary bedroom, a kitchen, and a guest room. The area of the secondary bedroom should be no less than 15% of the total area.
2. Xiao Ming's mother enjoys cooking, so the kitchen area should be no less than 5% of the total area.
3. To ensure good lighting, the master bedroom and secondary bedroom should be placed on the south side with windows.
4. To make it convenient to care for Xiao Ming's grandparents when they occasionally visit, the distance between the master bedroom door and the guest room door should be no more than 3 meters.
5. Other functional areas may be designed and arranged reasonably according to the available space.

Drawing Requirements

1. Use a ruler to draw each room clearly and distinctly on the floor plan.
2. Accurately and clearly label the dimensions of each room on the floor plan.

Task note: Although a bathroom was not explicitly listed in the written task prompt, it was treated as a necessary functional area in scoring because it is a conventional requirement for an apartment and was consistently considered in students' drawings and discussions.

Supplementary Figure S1. *Original Spatial Boundary Diagram for the Apartment Spatial Design Task*

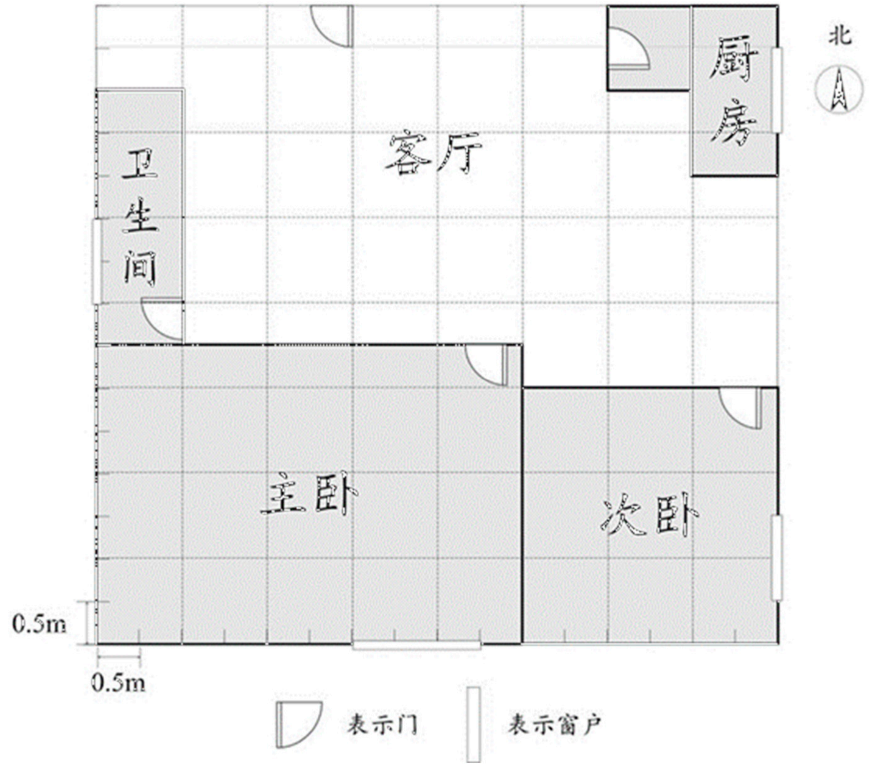

Note. The task was administered in Chinese. The original Chinese spatial-boundary diagram is reproduced here to preserve the task format presented to students. Chinese labels in the diagram are defined as follows: 卫生间 = bathroom; 客厅 = living room; 厨房 = kitchen; 主卧 = master bedroom; 次卧 = secondary bedroom; 北 = north; 表示门 = door; 表示窗户 = window.

Supplementary Table S1. *Structural Equivalence and Baseline Performance of the Parallel Collaborative Tasks*

Task version	Core design context	Shared structural demands	Pretest performance, *M* (*SD*)	Expert-rating profile
Xiao Ming's apartment	Apartment spatial design	Area-proportion, lighting-orientation, door-distance, spatial-boundary, and additional functional-area planning constraints	35.50 (6.03)	4, 4, 5, 4
Campus sports event	Campus venue design	Proportion/area, distance/position, orientation/boundary, functional-area arrangement, and venue circulation-planning constraints	35.25 (4.57)	4, 4, 5, 4

Note. Expert-rating profile is reported in the following order: mathematical structural complexity, spatial reasoning load, task openness, and integrated cognitive demand. Ratings were completed independently by three mathematics education experts on a five-point scale, with higher scores indicating greater demand: 1 = very low, 2 = low, 3 = moderate, 4 = high, and 5 = very high. Experts reviewed the task texts, spatial-boundary diagrams, design requirements, and rating instructions before rating. Values in the expert-rating profile are mean ratings; all standard deviations were 0.00. The identical expert-rating profiles and comparable pretest performance support the structural comparability of the two parallel task versions.

Supplementary Table S2. *Operational Definitions, Decision Rules, and Examples of Accountable Talk Codes*

Code	Code label	Operational definition	Decision rule	Example
1.1	Simple repetition	Repeats, restates, or locally continues words, numbers, objects, or proposals from the previous turn without adding a new idea.	Use when the turn primarily maintains interactional continuity without adding a constraint, explanation, evaluation, or reason.	A: "Xiao Ming's bed goes here." B: "Goes here."
1.2	Socialized cognitive alignment	Responds to a peer's idea or proposal by expressing agreement, acceptance, disagreement, or reservation, thereby establishing shared attention.	Use when the turn primarily signals a stance toward a peer's idea or focuses attention on that idea, rather than merely repeating the previous turn or providing a substantive evaluation of the proposal.	A: "The kitchen needs to be this big." B: "I disagree."
1.3	Elaboration/refinement	Adds details, conditions, constraints, or explanations that make an existing idea more specific.	Use when the turn extends or refines a previous expression rather than introducing a separate proposal.	A: "Let's put the kitchen near the entrance." B: "And closer to the dining room."
2.1	Mathematical fact stating	States task-related facts, conditions, data, proportions, areas, or distances.	Use when the turn invokes given or known information without proposing, evaluating, or reasoning about a solution.	"The secondary bedroom cannot be less than 15% of the total area."
2.2	Solution proposal	Proposes a spatial arrangement, layout plan, design choice, or problem-solving step.	Use when the turn specifies where to place, how to draw, how to design, or what solution to adopt.	"Let's put the kitchen next to the entrance."
2.3	Mathematical evaluation	Evaluates a proposal based on area, proportion, distance, spatial constraints, or reasonableness.	Use when the turn judges whether a proposal satisfies task constraints or is mathematically reasonable.	A: "Let's make the bedroom 2 meters wide." B: "That is too narrow."
3.1	Spatial reasoning	Reasons about geometric, spatial, or quantitative relationships.	Use when the turn explains a relationship or inference rather than merely stating a fact or proposing a layout.	"It is 4 meters long and 3 meters wide, so the room is 12 square meters."
3.2	Contextual application	Uses everyday experience, user needs, or functional considerations to explain a spatial arrangement.	Use when the turn connects the design with real-world use or practical needs.	"If the grandparents come to stay, the guest room should be closer to the master bedroom so it is easier to take care of them."
0	Non-AT	Does not constitute a valid task-related discourse function within the coding framework.	Use for off-task, unclear, purely managerial, or noise-related turns.	"Wait, I need to find my pen."

Note. AT = accountable talk. The coding framework operationalized AT as a set of turn-level discourse codes. Code labels are retained to indicate the specific coding categories used in manual coding. Code 0 indicates non-AT turns and was used for manual coding quality control; it was excluded from the main analyses. Short or generic responses without task-relevant mathematical, spatial, or explanatory content (e.g., "Ah?" or a stand-alone "Why?") were not coded as reasoning-oriented talk; they were assigned to lower-level social-alignment, repetition, or non-AT categories according to their interactional function.

Supplementary Table S3. *fNIRS Channel Mapping, Preprocessing, and Quality Control*

Panel A. fNIRS channels, coordinate axes, regions of interest, and channel group grouping

Channel	X	Y	Z	AAL label	BA label	Channel Group
1	20.15	58.2	57.59	Frontal_Sup_R	Right-BA9	C3 VAN
2	−11.03	61.43	58.44	Frontal_Sup_Medial_L	Left-BA8	C3 VAN
3	1.95	71.55	46.14	Frontal_Sup_Medial_L	Left-BA9	C1 CEN
4	35.2	63.15	39.44	Frontal_Mid_R	Right-BA9	C1 CEN
5	20.56	75.84	29.94	Frontal_Sup_R	Right-BA10	C1 CEN
6	34.77	73.56	16.81	Frontal_Sup_R	Right-BA10	C1 CEN
7	−27.17	67.69	42	Frontal_Sup_L	Left-BA9	C1 CEN
8	−14.68	78.93	27.99	Frontal_Sup_L	Left-BA10	C1 CEN
9	4.28	83.81	18.63	Frontal_Sup_Medial_R	Right-BA10	C1 CEN
10	19.52	82.98	3.52	Frontal_Sup_R	Right-BA10	C1 CEN
11	−25.54	79.94	13.86	Frontal_Sup_L	Left-BA10	C1 CEN
12	−10.16	85.08	1.91	Frontal_Sup_Medial_L	Left-BA10	C1 CEN

Channel	X	Y	Z	AAL label	BA label	Channel Group
13	−77.17	−25.6	50.99	SupraMarginal_L	Left-BA40	C2 LCN
14	−68.71	5.96	50.66	Precentral_L	Left-BA6	C3 VAN
15	−79.07	−11.12	38.11	Postcentral_L	Left-Prim Sensory	C3 VAN
16	−80	−44.62	36.65	SupraMarginal_L	Left-BA39	C2 LCN
17	−84.96	−28.04	24.06	SupraMarginal_L	Left-BA40	C2 LCN
18	−82.82	−46.33	4.83	Temporal_Mid_L	Left-BA21	C2 LCN
19	−70.49	19.61	35.96	Frontal_Inf_Oper_L	Left-BA44	C2 LCN
20	−78.59	3.01	23.21	Postcentral_L	Left-BA6	C3 VAN
21	−84.39	−11.59	7.87	Temporal_Mid_L	Left-BA21	C2 LCN
22	−86.3	−30.57	−11.07	Temporal_Mid_L	Left-BA21	C2 LCN
23	−77.01	14.54	7.09	Frontal_Inf_Oper_L	Left-BA44	C2 LCN
24	−79.98	2.25	−5.52	Temporal_Mid_L	Left-BA22	C2 LCN

Panel B. Preprocessing and quality-control parameters

Parameter	Operational specification
Device	Artinis Brite24.
Sampling rate	10 Hz.
Main hemodynamic signal	Oxygenated hemoglobin (HbO).
Baseline	A 4-min baseline rest period was collected.
Signal conversion	Raw intensity signals were converted to optical density before subsequent preprocessing.
Motion artifact correction	Wavelet-based motion artifact correction was applied.
Band-pass filter	0.01–0.5 Hz.
Global component removal	PCA regression was used to remove global components.
PCA threshold	Cumulative explained variance = 0.75.
Baseline correction	Baseline correction was applied before IBS computation.
IBS estimation	Wavelet transform coherence (WTC).
IBS frequency range	0.02–0.08 Hz.
Pairwise coherence	Pairwise same-channel coherence was computed among group members.
Channel Group aggregation	Channel-level coherence was averaged within member pairs and then aggregated to the group and channel group levels.
Record-level quality control	Records with a low-quality-channel proportion below 1/6 were retained.
Channel-level quality control	Within retained records, low-quality channels were excluded at the channel level and were not entered into WTC estimation or Channel Group-level averaging.
Handling of missing/low-quality channels	Channel Group-level IBS was computed from the remaining valid channels within each functional Channel Group. Low-quality channels were not replaced by adjacent channels in the main analysis.

Note. fNIRS = functional near-infrared spectroscopy; AAL = Automated Anatomical Labeling; BA = Brodmann area; IBS = inter-brain synchrony; WTC = wavelet transform coherence; PCA = principal component analysis. C1 CEN = Central Executive Channel Group, channels 3–12; C2 LCN = Language Communication Channel Group, channels 13, 16-19, and 21-24; C3 VAN = Visuospatial-Action Channel Group, channels 1, 2, 14, 15, and 20.

Supplementary Table S4. *Scoring Framework for the Apartment Spatial Design Task*

Scoring domain	Focus	Observable evidence in the submitted design
Functional-area planning	Inclusion of required functional areas and reasonable planning of additional spaces.	Required areas such as a master bedroom, secondary bedroom, guest room, kitchen, and bathroom; reasonable arrangement of a living room and other additional spaces.
Quantitative relationships and calculations	Accurate treatment of area proportions and dimensional relationships.	A secondary-bedroom area of at least 15% of the total area; a kitchen area of at least 5% of the total area; necessary dimension and area calculations.
Satisfaction of spatial constraints	Translation of contextual requirements into spatial-layout conditions.	South-side placement and lighting conditions for the master and secondary bedrooms; no more than 3 m between the master bedroom door and guest room door; compliance with the spatial boundary.
Door, window, and spatial relationships	Appropriate coordination of doors, windows, and functional spaces.	Reasonable alignment of room functions, doors, windows, circulation, and use.
Graphical representation and labeling	Clear representation of the proposed design.	Room boundaries, dimensions, areas, and other necessary labels; consistency between labels and the submitted drawing.
Overall design reasonableness	Construction of a feasible solution satisfying multiple requirements.	Functional completeness, spatial relationships, usability, and coherence of the overall layout.

Note. A task-specific additive rubric with a maximum score of 50 points was used. The scoring items were derived directly from the task requirements. Although a bathroom was not explicitly listed in the written prompt, it was treated as a necessary functional area in scoring because it is a conventional requirement for an apartment and was consistently considered in students' drawings and discussions. A living room and other additional spaces could be arranged according to the remaining available area. To preserve the usability of the scoring instrument for future research, the item-level scoring key is not reproduced here. The detailed scoring manual is available from the authors upon reasonable request.

Supplementary Table S5. *Full Model Results With 95% Confidence Intervals*

Panel A. RQ1 and RQ2 model results

RQ	Outcome	Effect	Estimate [95% CI]	*SE*	*p*	FDR-adjusted *p*	Level
RQ1	AT turn count	Task performance	19.889 [6.373, 33.405]	6.595	0.005	0.014	Group × Task
RQ1	Total AT duration	Task performance	0.773 [−58.095, 59.640]	28.752	0.979	0.979	Group × Task
RQ1	Proportion of AT duration	Task performance	0.042 [−0.006887, 0.091]	0.024	0.089	0.112	Group × Task
RQ1	Mean AT turn duration	Task performance	−0.472 [−0.870, −0.074]	0.195	0.022	0.036	Group × Task
RQ1	AT turn density	Task performance	2.699 [1.524, 3.874]	0.575	< 0.001	< 0.001	Group × Task
RQ1	AT turn density	Relative time	−0.436 [0.576, −0.296]	0.071	< 0.001	—	Window
RQ1	AT turn density	Task performance	2.229 [1.924, 2.534]	0.156	< 0.001	—	Window
RQ1	AT turn density	Relative time × task performance	−0.139 [−0.278, 0.000700]	0.071	0.051	—	Window
RQ1	AT turn density	Linear time interaction model comparison	$\chi^2(1) = 3.805$	—	0.051	—	Window
RQ1	AT turn density	Natural−spline time model comparison	$\chi^2(5) = 47.885$	—	< 0.001	—	Window
RQ2	IBS (standardized)	Task performance × Channel Group model comparison	$\chi^2(2) = 6.515$	—	0.038	—	Group × Task × Channel Group
RQ2	IBS (standardized)	Task performance slope in C1	0.061 [−0.283, 0.404]	0.173	0.726	0.726	Group × Task × Channel Group
RQ2	IBS (standardized)	Task performance slope in C2	−0.096 [−0.439, 0.248]	0.173	0.582	0.726	Group × Task × Channel Group
RQ2	IBS (standardized)	Task performance slope in C3	−0.520 [−0.864, −0.176]	0.173	0.003	0.010	Group × Task × Channel Group

RQ	Outcome	Effect	Estimate [95% CI]	*SE*	*p*	FDR-adjusted *p*	Level
RQ2	IBS	C2 vs. C1 Channel Group contrast	0.002207 [0.001092, 0.003323]	0.000569	< 0.001	< 0.001	Group × Task × Channel Group
RQ2	IBS	C3 vs. C1 Channel Group contrast	−0.001352 [−0.002468, −0.000237]	0.000569	0.018	0.018	Group × Task × Channel Group

Panel B. RQ2 Channel Group contrast and RQ3 model results

RQ	Outcome	Effect	Estimate [95% CI]	*SE*	*p*	FDR-adjusted *p*	Level
RQ2	IBS	C2 vs. C3 Channel Group contrast	0.003559 [0.002444, 0.004675]	0.000569	< 0.001	< 0.001	Group × Task × Channel Group
RQ3	IBS	AT turn density	0.001783 [0.001011, 0.002555]	0.000394	< 0.001	< 0.001	Window × Channel Group
RQ3	IBS	AT turn density × task performance	0.000731 [0.000232, 0.001231]	0.000254	0.004	0.006	Window × Channel Group
RQ3	IBS	AT turn density slope at low task performance	0.000537 [−0.000581, 0.001654]	0.000570	0.346	0.346	Window × Channel Group
RQ3	IBS	AT turn density slope at mean task performance	0.001268 [0.000442, 0.002094]	0.000421	0.003	0.004	Window × Channel Group
RQ3	IBS	AT turn density slope at high task performance	0.001999 [0.001216, 0.002783]	0.000399	< 0.001	< 0.001	Window × Channel Group
RQ3	Mean IBS	Aggregated AT density	0.001190 [−0.000842, 0.003222]	0.000947	0.230	0.345	Aggregated Group × Task × Channel Group
RQ3	Mean IBS	Aggregated AT density × task performance	0.001750 [0.000981, 0.002519]	0.000359	< 0.001	< 0.001	Aggregated Group × Task × Channel Group
RQ3	Mean IBS	Aggregated cognitive AT density × task performance	0.002342 [0.001214, 0.003470]	0.000526	< 0.001	< 0.001	Aggregated Group × Task × Channel Group

Note. AT = accountable talk; IBS = inter-brain synchrony; FDR = false discovery rate. Values in brackets are 95% confidence intervals. Model-comparison rows report likelihood-ratio χ^2 tests, for which *SE*s and confidence intervals are not defined. FDR-adjusted *p* values are Benjamini-Hochberg adjusted within the corresponding outcome, contrast, or slope families. Aggregated robustness models use HC1 team-cluster robust *SE*s. Exploratory phase-specific RQ2 results, where reported, should be interpreted cautiously.

Supplementary Table S6. *PCA Regression Sensitivity Summary Across Explained-Variance Thresholds*

PCA threshold	Number of PCs removed	Median cumulative explained variance removed
PCA50	Median = 2; IQR = 0; Range = 1–4	59.07%
PCA60	Median = 2; IQR = 1; Range = 1–5	66.45%
PCA75	Median = 4; IQR = 2; Range = 1–8	77.94%

Note. PCA regression was performed separately for each participant-session using the 24-channel HbO matrix. Principal components were ordered according to explained variance, and the minimum number of leading components required to reach the specified cumulative explained-variance threshold was regressed out. Values represent the median number of removed components and the median cumulative explained variance removed across participant-session records.

Supplementary Table S7. *Sensitivity Analyses of IBS Findings Across PCA Explained-Variance Thresholds*

Note. Sensitivity analyses were conducted using alternative PCA regression thresholds (PCA50, PCA60, and PCA75). All other preprocessing and analytical procedures were held constant, including motion correction, filtering parameters, channel configuration, WTC estimation, temporal-window construction, Group IBS calculation, and statistical models. The PCA75 condition corresponds to the preprocessing specification used for the primary analyses reported in the manuscript.

Panel A. RQ2 Sensitivity Analysis: Performance × Channel-Group Association

PCA threshold	Overall interaction	C3 slope (β)	BH-adjusted p (C3)	Interpretation
PCA75	$p = 0.0385$	−0.5199	$p = 0.0104$	C3 negative association significant after BH correction
PCA60	$p = 0.0314$	−0.4260	$p = 0.0611$	Direction preserved; BH-corrected significance not reached
PCA50	$p = 0.0088$	−0.5399	$p = 0.0069$	C3 negative association significant after BH correction

Note. Additional channel-group results: C1 and C2 effects were not statistically significant across PCA thresholds, and their small point estimates showed less stable directional patterns.

Panel B. RQ3 Sensitivity Analysis: Accountable Talk–IBS Associations

Analysis level	Effect	PCA75	PCA60	PCA50
Window	AT main effect	Positive, $p < 0.001$	Positive, $p = 0.013$	Positive, $p = 0.002$
Window	AT × task-performance interaction	Positive, $p = 0.004$	Positive, $p = 0.213$	Positive, $p < 0.001$
Aggregate	AT main effect	Positive, $p = 0.230$	Positive, $p = 0.946$	Positive, $p = 0.523$
Aggregate	AT × task-performance interaction	Positive, $p < 0.001$	Positive, $p = 0.283$	Positive, $p = 0.135$

Summary. Across PCA thresholds, the direction of the focal IBS associations was generally consistent. The temporal-window AT main effect remained statistically significant across all three PCA specifications. The AT × task-performance interaction remained positive, although statistical significance varied across preprocessing specifications. Aggregate-level effects showed greater sensitivity to PCA threshold choice and analytical aggregation level.
